# Nanostructured Sr-Doped Hydroxyapatite: A Material with Antimicrobial Potential

**DOI:** 10.3390/nano15211651

**Published:** 2025-10-29

**Authors:** Miljana Mirković, Aleksandra Sknepnek, Ana Kalijadis, Aleksandar Krstić, Marija Šuljagić, Marko Perić, Ljubica Andjelković

**Affiliations:** 1 Department of Materials, “VINČA” Institute of Nuclear Sciences—National Institute of the Republic of Serbia, University of Belgrade, Vinča, 11351 Belgrade, Serbia; ana.kalijadis@vin.bg.ac.rs; 2 Institute of Food Technology and Biochemistry, Faculty of Agriculture, University of Belgrade, Nemanjina 6, Zemun, 11080 Belgrade, Serbia; aleksandras@agrif.bg.ac.rs; 3 Department of Physical Chemistry, “VINČA” Institute of Nuclear Sciences—National Institute of the Republic of Serbia, University of Belgrade, 11000 Belgrade, Serbia; aleksandar.krstic@vin.bg.ac.rs; 4 Institute of Chemistry, Technology and Metallurgy, Institute of National Importance for the Republic of Serbia, University of Belgrade, Njegoševa 12, 11000 Belgrade, Serbia; marija.suljagic@ihtm.bg.ac.rs (M.Š.); ljubica@chem.bg.ac.rs (L.A.); 5 Department of Radiochemistry, “VINČA” Institute of Nuclear Sciences—National Institute of the Republic of Serbia, University of Belgrade, 11000 Belgrade, Serbia; markoperic1983@gmail.com

**Keywords:** hydroxyapatite, nanostructure, antimicrobial properties, strontium

## Abstract

This research investigated the feasibility of producing strontium-doped nanocrystalline hydroxyapatite (SrHAp) through an environmentally benign synthesis approach and evaluated the antimicrobial activity of the resulting material. The synthesized nanomaterial was subjected to comprehensive characterization. The antimicrobial efficacy of SrHAp was tested against Gram-positive and Gram-negative bacterial strains. X-ray diffraction (XRD) analysis in combination with Fourier-transform infrared (FT-IR) spectroscopy confirmed the successful formation of pure monocrystalline SrHAp. The scanning electron microscopy (SEM) examination revealed two predominant morphological structures: nanorods and prismatic configurations of the SrHAp. Transmission electron microscopy (TEM) demonstrated that the rod-like SrHAp nanocrystals aggregate into elongated grain structures with a size of about 25 nm × 10 nm. Inductively coupled plasma-optical emission spectroscopy (ICP-OES) analysis confirmed the presence and quantification of the concentrations of calcium, strontium, and phosphorus, while confirming the expected calcium–phosphorus ratio characteristic of hydroxyapatite. The study established that the positive surface charge of the material, with a point of zero charge near pH 10, is essential for its antimicrobial efficiency. These results suggest that SrHAp nanomaterials hold promise for biomedical applications, particularly as antimicrobial coatings for implants and scaffolds for bone tissue, where the prevention of infection is critical. Overall, despite its selective and material quantity-dependent antimicrobial efficacy, environmentally friendly synthesized SrHAp can be successfully applied as an effective controller of targeted microbial contamination, especially of Gram-positive bacterial species *S. aureus*, *L. monocytogenes*, *S.* Enteritidis, and *A. baumanii.*

## 1. Introduction

Antimicrobial resistance is one of the most pressing global health challenges of the 21st century. This serious threat to public health arises when microorganisms develop resistance to previously effective antimicrobial drugs, leading to around 4.95 million deaths from bacterial infections caused by resistant strains in 2019 alone [1,2,3,4,5]. To address the escalating obstacles of antimicrobial resistance, the World Health Organization (WHO) has given strategic priority to identifying and validating novel therapeutic interventions that can serve as effective replacements for conventional antibiotic protocols [6]. Nanoparticulate calcium phosphates, especially hydroxyapatite (HAp), have become a groundbreaking material in biomedical research [7,8,9,10]. As a synthetic counterpart to biological apatite, HAp has demonstrated remarkable versatility in pharmaceuticals, dentistry, and medical applications [11,12,13]. Notably, extensive research has brought to light its strong antimicrobial effect as well as other critical properties [12,14,15]. Over decades of scientific work, researchers have developed a variety of multifunctional HAp materials that not only combat bacterial growth but also offer additional benefits such as osteoconductive and osteoinductive characteristics [15,16,17,18,19]. The ability of the material to suppress microbial proliferation, combined with its regenerative properties, makes HAp a particularly promising agent in various medical fields. Despite HAp’s limited solubility, its remarkable ability to maintain high lattice strains and accommodate foreign ions has revolutionized materials science. Strategic doping of HAp nanoparticles with various elements has proven to be a sophisticated approach to modifying its chemical, physical, and biological properties [12,14,15,16,17,18,19,20]. Researchers have explored various dopants such as Al^3+^, Fe^3+^, and Ni^2+^ to modify the hexagonal structure of HAp in order to achieve antimicrobial, biocompatible, and bone regenerative properties [21,22,23,24,25]. Magnetic metal ions like Fe^2+^ and Co^2+^ have been incorporated into HAp nanostructures for applications in magnetic resonance imaging, targeted drug delivery, and hyperthermia [26,27]. Similarly, Ni^2+^, Zn^2+^, and silica ions have shown effective antibacterial properties as HAp dopants against *Escherichia coli*, *Candida albicans*, and *Pseudomonas aeruginosa* [22,28,29]. Particularly important is the targeted introduction of ions such as Mg^2+^, Sr^2+^, and Zn^2+^ to improve antimicrobial properties, which have become an important issue in reconstructive and regenerative medicine [30,31,32,33,34]. The hexagonal structure of HAp enables precise ion substitution at the Ca1 and Ca2 positions. This allows researchers to specifically develop improved antimicrobial capabilities while maintaining the structural integrity of the material. This ion-doping strategy fundamentally changes interactions of HAp with biological systems. Doped nanoparticles show significantly different cellular responses compared to undoped variants [14]. The ability to systematically modify the antimicrobial potential of HAp represents a breakthrough in the development of modern materials with customized biological performance.

The research focuses on HAp doped with 20 atomic percent strontium, which remains within the limits of biocompatibility. Previous research has demonstrated that remineralizing formulations with 20% strontium-doped hydroxyapatite (SrHAp) materials have significantly stronger antibacterial activity compared to 10% Sr-doped formulations [35,36].

This research focuses on environmentally compatible synthesis methods for Sr-doped HAp nanomaterials. Instead of the commonly used nitrates and aggressive sulfuric acid, the study utilizes acetate precursors as calcium and strontium sources in addition to hydrogen phosphate.

The novelty of this research lies in the fact that materials conventionally used as biocompatible materials can also serve as antimicrobial agents. It is important to note that there is limited literature on the antimicrobial properties of hydroxyapatite material solely doped with strontium ions and with precisely defined stoichiometry. Most research has focused on silver-containing materials, which are well-studied and noted for their antimicrobial properties [37,38]. Moreover, in contrast to our study, many existing studies focus on other antimicrobial agents, such as copper, magnesium, and zinc. These agents can also be integrated into the hydroxyapatite (HAp) structure and have been thoroughly investigated for their antimicrobial properties. Additionally, some research explores composites made from nanomaterials and organic polymers [14]. Our research specifically investigates strontium-doped hydroxyapatite (SrHAp) materials with defined stoichiometric and morphological characteristics.

## 2. Materials and Methods

### 2.1. Synthesis Procedure of SrHAp Material

The SrHAp material was synthesized by the chemical precipitation method, with the nominal chemical composition SrCa_4_(PO_4_)_3_OH [39]. An aqueous solution of sodium dihydrogen phosphate (NaH_2_PO_4_, p.a. ≥98%, Kemika, Zagreb, Croatia) was prepared by dissolving 0.7452 g in 25 cm^3^ in distilled water. Then, solutions containing 1.1388 g of calcium acetate (Ca(CH_3_COO)_2_ 99.99%, RPE, Carlo Erba, Cornaredo, Italy) and 0.3702 g of strontium acetate (Sr(CH_3_COO)_2_, p.a. Sigma Aldrich, St. Louis, MO, USA) were each dissolved in 25 cm^3^ volume of distilled water and added dropwise. In order to form hydroxyapatite structure, 10 cm^3^ of ammonium hydroxide solution (NH_4_OH, p.a. purity, 25%, Carlo Erba) was introduced. The reaction temperature was maintained at 90 °C for 2 h, while the pH value of the obtained mixture was 11.6 (pH checker, Labware, Wilmington, DE, USA). The mixture was allowed to age overnight. The precipitate was separated from solution by centrifugation, rinsed with deionized water three times, and washed once in ethanol. The obtained powder was dried overnight at 70 °C under ambient air conditions.

The synthesized nano-SrHAp is subjected to comprehensive structural and microstructural analysis, using X-ray diffraction (XRD), Fourier-transform infrared (FT-IR) spectroscopy, scanning electron microscopy (SEM), and transmission electron microscopy (TEM). The strontium content was determined using inductively coupled plasma optical emission spectrometry (ICP-OES). Antimicrobial analysis was performed to assess antimicrobial properties of the material—a decisive ability in the fight against pathogenic microorganisms in times of increasing antibiotic resistance.

### 2.2. Characterization Analyses

The X-ray diffraction—XRD—method was used for the phase and structural analyses of the material obtained. X-ray powder diffractometer Rigaku Ultima IV (Rigaku, Tokyo, Japan) equipped with K*α*_1,2_ radiation, using a generator voltage (40.0 kV) and a generator current (40.0 mA), with analog detector. The samples were placed in a monocrystalline silicon carrier and scanned in the range of 5 to 60° 2*θ* in a continuous scan mode with a step size of 0.02° and a scan rate of 5°/min. The PDXL2 software (Version 2.8.4.0) was utilized for phase identification and Rietveld profile refinement [40], which is supported by a crystallographic structure database from ICDD [41]. The DB card number 01-075-8113 and 01-089-4405 was used for phase identification and Rietveld profile refinement of the obtained structure.

The IR spectrum was recorded using the ATR technique with a Nicolet IS35 Thermofisher Scientific FTIR spectrometer (Waltham, MA, USA).

For ICP-OES analysis, samples were prepared using acid-wet digestion. A 0.2 g sample was weighed into glass vessels and digested with 5 mL concentrated HNO_3_ and 10 mL concentrated HCl for 15 min at 800 °C. After digestion, the solution was cooled and filtered through 0.45 μm filter paper. The filtered solution was then transferred to a 100 mL volumetric flask and diluted to volume with deionized water.

Quantification of Ca, Sr, and P was performed using a Thermo Fisher Scientific model 7400 Duo ICP-OES spectrometer (Waltham, MA, USA). Analytical standards for instrument calibration were prepared from single-element standard solutions of Ca, Sr, and P (100 ppm concentration). Results are presented as mass percentages.

The morphology of the SrHAp was determined using the SEM (JEOL JSM-6390 LV, Tokyo, Japan). The accelerating voltage in the SEM was in the range of 20–30 kV. The TEM analysis was performed using a JEOL JEM-1400 Plus Electron microscope (Tokyo, Japan) with a voltage of 120 kV and a LaB_6_ filament, at a magnification of 250,000×.

The pH drift method was applied to determine the pH values of the point of zero charge (pH_PZC_) [42]. The initial solutions contained sodium chloride (0.01 M, 25 cm^3^). The pH values between 2 and 12 were adjusted with sodium hydroxide (0.1 M) and hydrochloric acid (0.1 M) solutions. Then, 75 mg SrHAp was added. The final pH values were measured after 48 h at room temperature. The pHpzc values were determined where the pH_final_ vs. pH_initial_ curve crossed the line pH_initial_ = pH_final_.

Density Functional Theory (DFT) was used to investigate the structural and electronic properties of SrHAp. The structures of the investigated complexes were optimized at the LDA level of the theory [43]. The Zero-Order Regular Approximation (ZORA) [44,45,46] was used to account for relativistic effects. For Ca and Sr ions, the old-ZORA-TZVPP basis set [47] was used, and for all other atoms the relativistically recontracted version of the all-electron def2-TZVP Ahlrichs basis set [47]. In addition, the SARC/J auxiliary basis set [48] was used, which is a decontracted def2/J auxiliary set that is more accurate for relativistic calculations. In order to compensate for the negative charge, water was considered as a solute by a conductor-like polarizable continuum model (CPCM) [49]. The ORCA program package was used for all calculations [50].

### 2.3. Antimicrobial Activity

Five pathogenic microorganisms were selected to test the material, two Gram-positive bacteria (*Staphylococcus aureus* ATCC 25923 and *Listeria monocytogenes* ATCC 19111) and three Gram-negative bacteria (*Escherichia coli* ATCC 25922, *Salmonella enterica* serovar Enteritidis ATCC 13076, and *Acinetobacter baumannii* ATCC 19606). These species were selected to provide a representative panel of opportunistic strains associated with hospital infections (*S. aureus*, *E. coli*, and *A. baumannii*) as well as pathogenic bacteria of food origin (*L. monocytogenes* and *S.* Enteritidis) that can cause severe invasive infections especially in vulnerable patients. In addition, *A. baumannii* represents a pathogen with high prevalence of multidrug resistance in hospital environments [51].

To prepare the microorganisms for analysis, *S.* Enteritidis was cultivated on Müeller Hinton agar (MHA, HiMedia, Thane, Maharashtra, India) while *L. monocytogenes*, *S. aureus*, *E. coli*, and *A. baumannii* were cultivated on Tryptone soy agar (TSA HiMedia). All bacteria were cultivated at 37 °C for 24 h. To prepare the inoculums, the colonies obtained were suspended in the corresponding broths, i.e., Malt broth (MB, Torlak, Belgrade, Serbia), Müeller Hinton broth (MHB, HiMedia) or Tryptone soy broth (TSB, HiMedia), using a McFarland densitometer DEN-1 (Biosan, Riga, Latvia) to adjust the initial cell concentration to ~10^5^ colony-forming units per ml (CFU/mL). The exact initial number of viable cells was determined by a total plate count assay [49].

Before antimicrobial analysis, the SrHAp material was weighed (50 mg and 100 mg). The material was placed in test tubes, then sterilized in an autoclave at 121 °C for 15 min [52]. To determine the antimicrobial activity of SrHAp, 1 mL suspension of microorganisms was added to the prepared samples in glass tubes (dimensions: 100 mm × 8 mm) closed with non-hermetic stoppers, leaving approximately 90 mm of headspace filled with atmospheric air. To ensure homogeneity and direct contact of material with cells, the suspension with powder SrHAp material was vortexed for 30 s. Incubation was conducted at 37 °C for 24 h in static conditions. Microbial broths without SrHAp sample were treated in the same manner and served as positive controls. After incubation, samples were vortexed for 60 s using vortex mixer in order to dissociate cells from the material surface [53]. Viable cells from bacterial suspension were counted by preparing serial dilutions in normal saline and plating on the appropriate agar for further incubation for 24 h. Analyses were performed in triplicates and results are expressed as mean viable cell counts ± standard deviation in log_10_ CFU/mL. Reduction (R) of viable cells was calculated as follows:

R (%) = (A − B)/A × 100%

where A represents the average number of viable bacterial cells in control sample, while B is the average number of viable bacterial cells of sample with the testing material [53]. After the reduction test, samples and controls were sonicated in an ultrasonic bath (Jeio Tech, UCP-02, Seoul, Republic of Korea) for 15 min to detach any potentially adhered bacteria. The resulting suspensions were serially diluted in saline, re-plated on agar, and after incubation, the colony counts compared with the initial results.

Statistical analysis was performed using GraphPad Prism 10.5 software. One-way analysis of variance (ANOVA) was used to assess differences between groups, and Tukey’s Honestly Significant Difference (HSD) test was used to determine statistically significant differences between group means at a significance level of *p* < 0.05 [52].

## 3. Results

### 3.1. X-Ray Diffraction (XRD) and Microstructural Analysis Results of SrHAp

**Figure 1:**
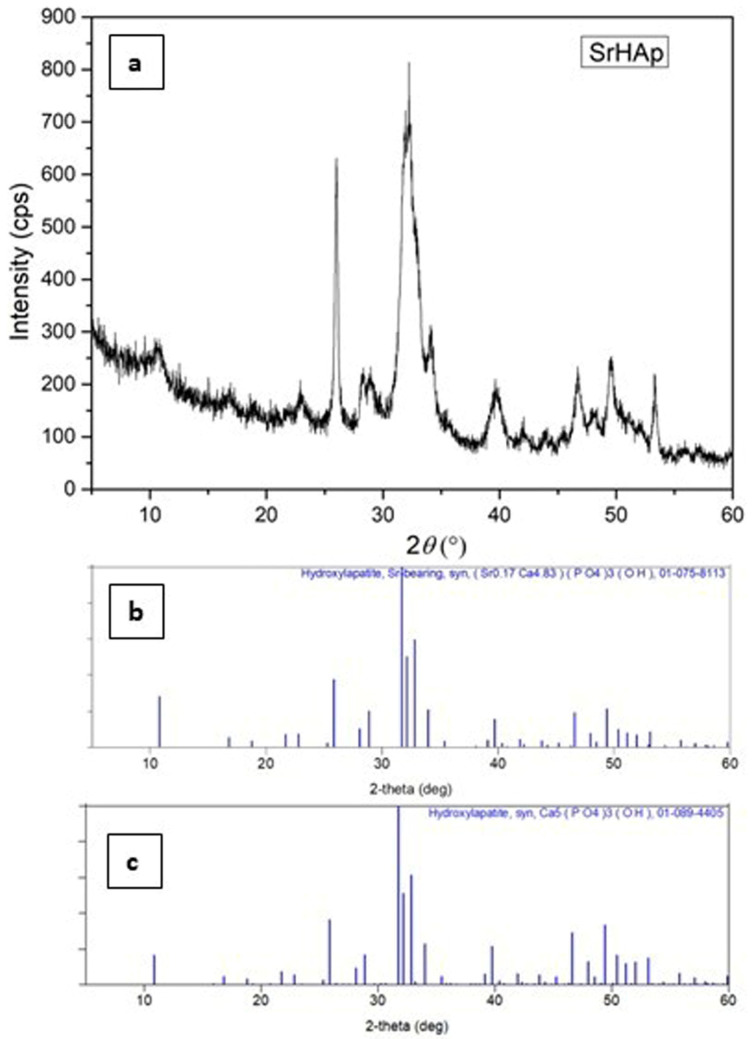
XRD patterns of (**a**) synthesized SrHAp material (experimental), (**b**) PDF card of Sr-bearing hydroxyapatite: 07-075-8113 and (**c**) PDF card of hydroxyapatite: 01-089-4405.

The results of the X-ray diffraction analysis are shown in Figure 1. The data confirm the successful synthesis of nanocrystalline strontium-doped hydroxyapatite by a solution-precipitation method. All detected diffraction peaks correspond to the strontium-containing hydroxyapatite phase, demonstrating the formation of a single-phase pure material. Structural analysis shows that the SrHAp composition matches the reference pattern (blue lines in Figure 1), confirming the hexagonal crystal symmetry within the P63/m space group.

The observed peak broadening, reduced intensity, and increased background signal indicate reduced crystallite dimensions as well as reduced structural order and crystallinity. These structural modifications result from strontium substitution at calcium sites, which causes lattice expansion due to the larger ionic radius of Sr^2+^ compared to Ca^2+^ [54]. While the crystallinity of the SrHAp sample decreases slightly, the hexagonal symmetry is preserved. The main reflections demonstrate subtle shifts to lower angles at approximately 31°, 28°, and 25° 2*θ*, confirming the incorporation of strontium into the hexagonal HAp framework [39,55].

Nanocrystalline materials exhibit characteristic diffraction patterns featuring peaks with significant full width at half maximum (FWHM) values [56]. Structural database limitations may restrict the precision of phase identification; therefore, the most compatible crystallographic reference card for our material is presented. No secondary phases were detected during the analysis. The calculated refined profile and structural parameters are summarized in Table 1.

The refined structural parameters confirm successful synthesis of SrHAp material with hexagonal structure, as evidenced by unit cell parameters of *a* = 9.41 Å and *c* = 6.92 Å, which correlate with previously reported values [54]. These lattice parameters verify successful partial substitution of Ca^2+^ by Sr^2+^ ions within the crystal structure [57]. The calculated crystallite size is approximately 49 Å, with no detectable stress or strain present in the crystal lattice. The refinement quality indicators demonstrate satisfactory results: the weighted profile R-factor (Rwp) of 6.24% falls well below the 15% threshold considered acceptable for hexagonal structure refinement, while the goodness-of-fit parameter (S factor) slightly exceeds 1.5, which remains acceptable given the nanocrystalline nature of the material.

### 3.2. Attenuated Total Reflectance Fourier Transform Infrared (ATR-FT-IR) Analysis Results of SrHAp

**Figure 2:**
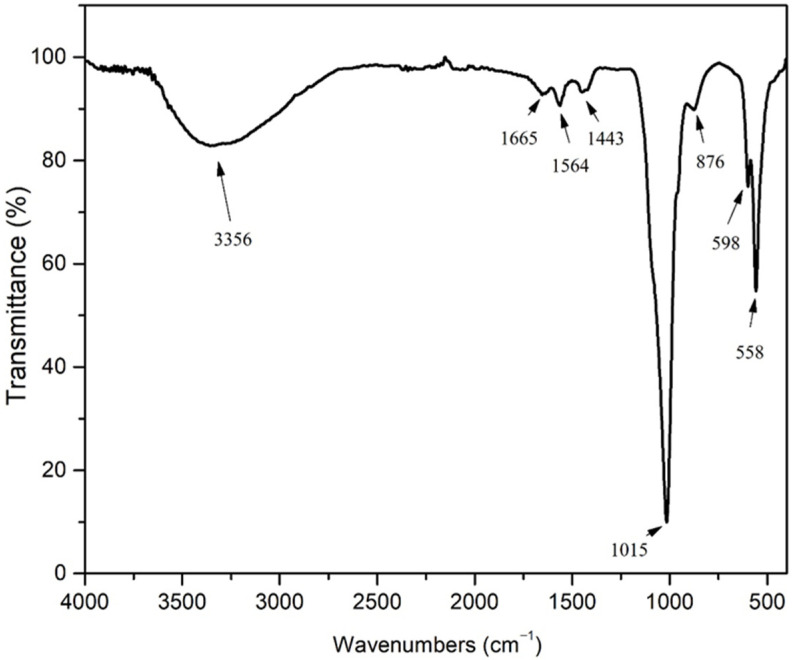
FT-IR spectrum SrHAp.

The FTIR spectrum (Figure 2) displays characteristic hydroxyapatite bands along with additional peaks due to common impurity ions (CO_3_^2−^, HPO_4_^2−^) typically found in such materials. In HAp materials, the band at approximately 3370 cm^−1^ arises from stretching vibrations of the OH^−^ group. The appearance of a band at 1665 cm^−1^ in the SrHAp sample indicates the presence of associated water molecules. Characteristic bands of the phosphate group (PO_4_^3−^) are observed in the range 598–1016 cm^−1^ [58,59,60]. The band at approximately 870 cm^−1^ corresponds to planar molecular vibrations of HPO_4_^2−^ [58]. The peak at 558 cm^−1^ is attributed to the asymmetric O-P-O bending deformation vibrations. The sharp *ν*_3_ signal at 1015 cm^−1^ represents asymmetric P-O stretching vibrations within the PO_4_^3−^ groups [61]. Weak peaks at 876 cm^−1^ and 1665 cm^−1^ are assigned to C-O bands, indicating carbonate impurity probably adsorbed by atmospheric exposure [62]. In the SrHAp sample, the bands at 1564 cm^−1^ and 1443 cm^−1^ correspond to vibrational modes of a predominantly “A-type” carbonate substitution, where CO_3_^2−^ ions replace OH^−^ ions in the hydroxyapatite structure, which is consistent with the XRD results shown in Figure 1 [39,63].

### 3.3. Inductively Coupled Plasma Optical Emission Spectroscopy (ICP-OES) Analysis

ICP-OES analysis was employed to verify the successful incorporation of strontium into the HAp structure. Table 2 presents both nominal and experimentally determined elemental concentrations.

The chemical formula of this compound is SrCa_4_(PO4)_3_OH, which can also be given as Sr_2_Ca_8_(PO4)_6_(OH)_2_ for the sake of simplicity. Based on the experimental results, the actual chemical composition of the SrHAp material can be represented as Sr_0.7_Ca_4.3_(PO_4_)_3_OH or equivalently as Sr_1.4_Ca_8.6_(PO_4_)_6_(OH)_2_. The primary aim was to achieve a partial substitution of Ca atoms by Sr atoms in the hydroxyapatite structure within the permissible limit of up to 15%, which was the synthesis goal of this study. It is important to note that in this case, the measurement uncertainty of the determined phosphorus concentration is approximately 1.20%, allowing the result to be expressed as 17.90 ± 1.20%. The analysis was performed in triplicate, and the reported value represents the mean of these replicates.

Table 3 presents the calculated nominal and experimental molar ratios for Ca/P and Ca/Sr. The experimental values for both molar ratios demonstrate close agreement with the nominal stoichiometric ratios derived from the theoretical formula. Furthermore, the experimentally determined Ca + Sr molar ratio is very close to the stoichiometric ratio of pure HAp (1.67). This agreement between experimental and nominal values is convincing evidence of successful strontium doping, which was confirmed by ICP-OES analysis.

### 3.4. Scanning Electron Microscopy (SEM) Analysis Results of SrHAp

**Figure 3:**
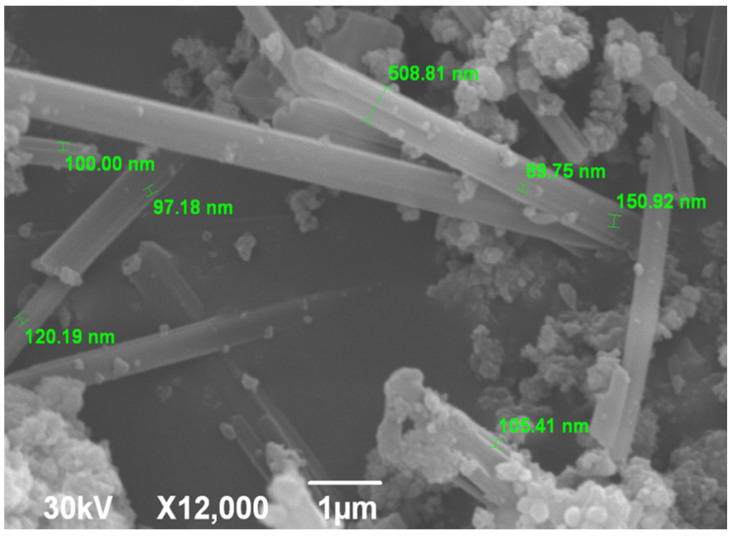
SEM microphotograph of SrHAp material.

SEM micrograph is shown in Figure 3. The image shows elongated rod-shaped morphologies resembling hexagonal prismatic SrHAp structures. These prismatic formations measure 5–7 μm in length and appear to be interconnected. Individual nanorods have a width of about 100 nm, while the interfaces between the agglomerated reach a thickness of up to 500 nm. All individual nanorods display uniform morphology that varies only in the dimensional parameters. This prismatic morphology of the Sr-doped HAp materials is related to the synthesis method used and is consistent with previously reported results [55,64]. Flake-like hexagonal crystals, characterized by small size and agglomeration, also represent this type of material, although their crystal growth mechanism differs from that of elongated crystalline forms [65].

### 3.5. Transmission Electron Microscopy (TEM) Results of SrHAp

**Figure 4:**
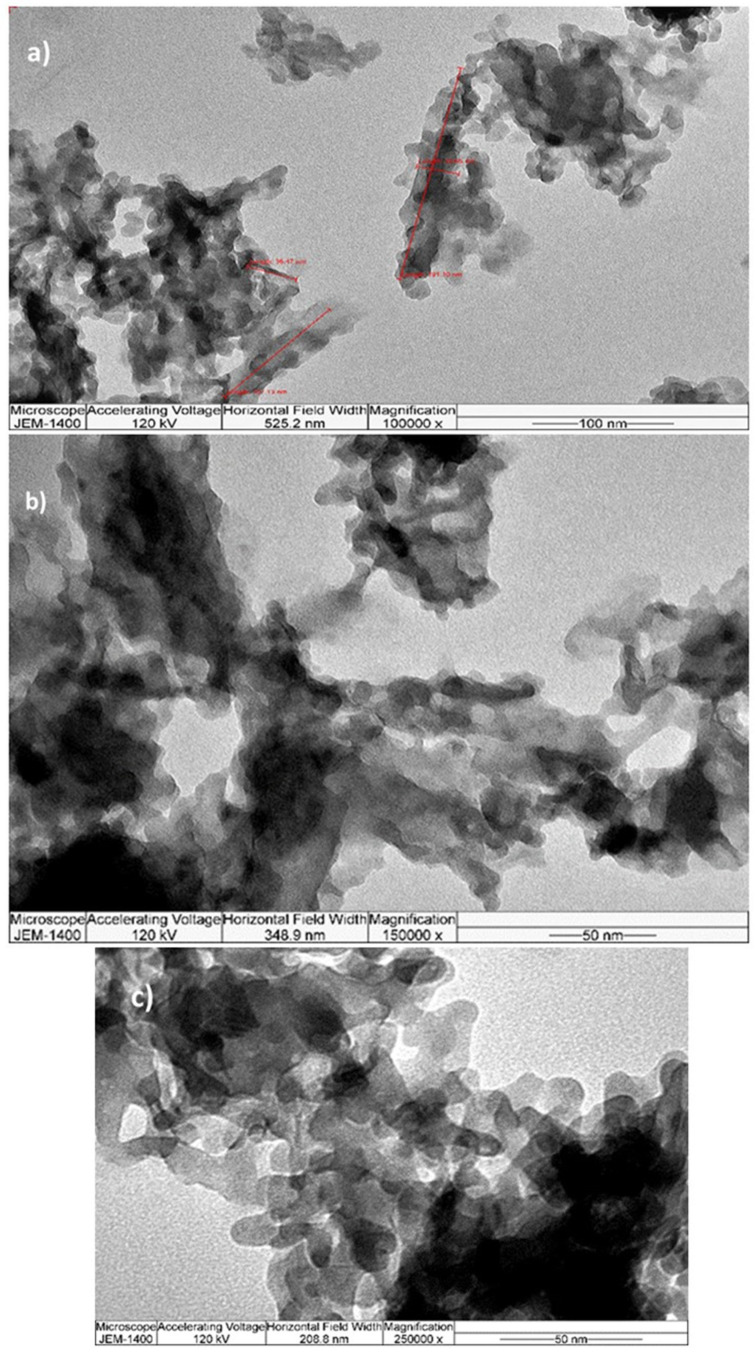
TEM microphotographs at different magnifications (**a**) 100 k, (**b**) 150 k, (**c**) 250 k.

TEM analysis was performed to comprehensively investigate the nanocrystalline SrHAp material. The TEM micrographs are shown in Figure 4. The images reveal rod-shaped nanocrystal geometry with different aggregation states. At lower magnification (Figure 4a,b), the crystals appear interconnected and form elongated grains or agglomerates with unidirectional growth, which is consistent with Sr-doped HAp materials with Sr content of 10–25% Sr [66]. The nanocrystals are 25 × 10 nm in size and have a relatively uniform morphology, as can be seen at higher magnification (Figure 4c). The calculated crystallite size is approximately 4.9 nm, indicating that each crystal consists of 2–5 crystallites separated by grain boundaries with no detectable strain or stress within the structure.

### 3.6. The Point Zero Charge (PZC) Analysis of SrHAp

**Figure 5:**
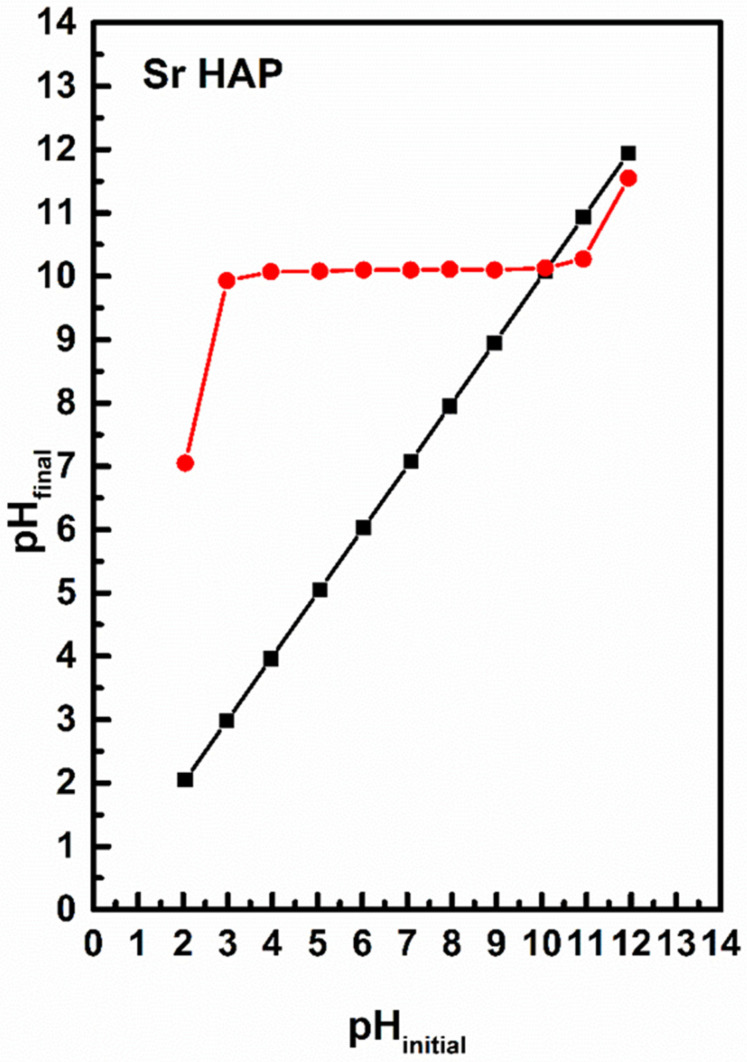
The pH_PZC_ values for the SrHAp sample (black line represents pH initial while red line represents pH final). The intersection of the black and red lines represents the pH_PZC_ of SrHAp material.

The pH_PZC_ value characterizes the acidity/basicity of the adsorbent and indicates the net surface charge of the material. The pH_PZC_ values obtained were relatively high and the results are shown in Figure 5. The pH_PZC_ value of SrHAp (red line) was around 10. This pH_PZC_ value improves particle–cell membrane interactions, and facilitates the penetration of material into bacterial cells [53]. The SrHAp material exhibits a positive surface charge at pH values below pH_PZC_, which promotes antimicrobial activity when the material surface is positively charged. Consequently, this material possesses favorable pH_PZC_ values that enable optimal antimicrobial efficacy at physiologic pH.

### 3.7. Density Functional Theory (DFT) Calculations

**Figure 6:**
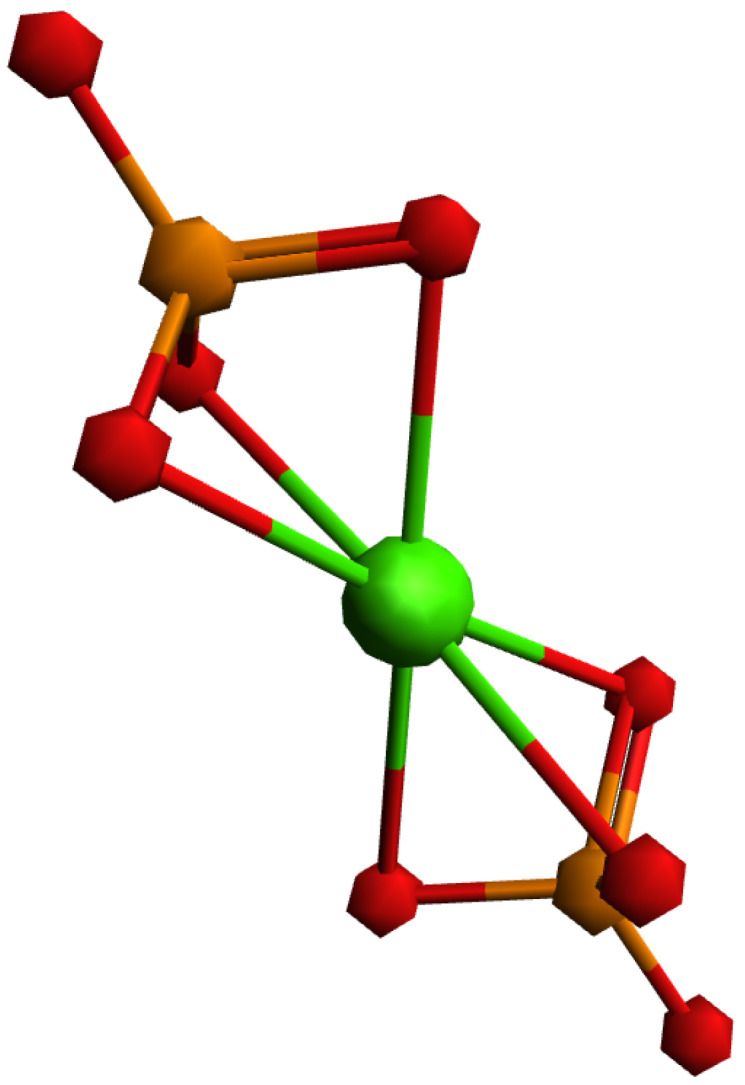
Investigated model system with general formula [M(PO_4_)_2_]^4−^, where central metal ion (Ca, Sr) is coordinated with two three-dentate phosphate ions.

**Figure 7:**
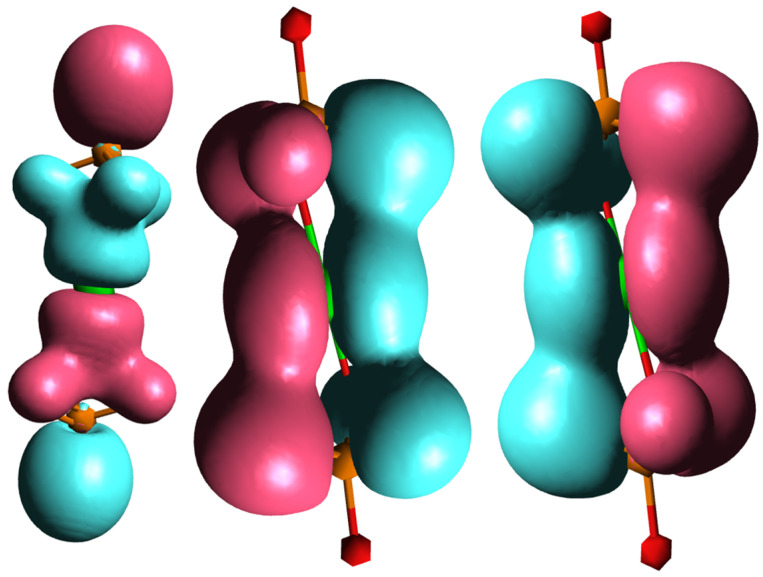
Molecular orbitals of investigated model system showing bonding interactions between symmetry-adapted orbitals of phosphate and *p* orbitals of central metal ion.

To explain the different behaviors of HAp and SrHAp, a simplified model was employed using DFT, as illustrated in Figure 6. The proposed model assumes that the central metal ion (Ca or Sr) coordinates with two phosphate ions, with each phosphate ion acting as a tridentate ligand, resulting in a coordination number of 6. Due to the tetrahedral geometry of the phosphate ion, the bond angles between metal and ligand deviate from the ideal octahedral symmetry. The bonding interactions in these models can be described by the overlap of the symmetry-adapted orbitals of the phosphate tetrahedra with the atomic orbitals of calcium or strontium (Figure 7).

Considering only the bonding interactions between the symmetry-adapted orbitals of the phosphate and the *p* orbitals of the metal ion, two interaction types are observed: frontal overlap and lateral overlap. This represents a simplified model system, since in hydroxyapatite there is actually an ionic bond between phosphate ions and calcium ions (Ca^2+^) which form the overall structure. The molecular orbitals of the phosphate ion determine its interactions with the calcium ions and determine its fundamental properties.

The comparison of the optimized geometries for the investigated model systems shows an extension of the metal–ligand bond in the strontium–phosphate system compared to the calcium–phosphate system. The strontium–phosphate model shows an average Sr-O bond length of 2.621 Å, while the calcium–phosphate model shows an average Ca-O bond length of 2.432 Å. This difference correlates with the enhanced ionic character of the strontium–phosphate bond, which results from the larger Sr^2+^ ionic radius and reduced charge density of the Sr^2+^ ion.

The analysis of atomic charge (Table 4) confirms these observations. In both model systems, there is a positive charge distribution on the metal ion and on the phosphorus atoms, with the metal ion having a higher positive charge than individual phosphorus atoms. However, the comparative analysis reveals greater positive charge accumulation on Sr than on Ca. Simultaneously, the phosphorus atoms in compound 1 display higher positive charges than those in compound 2. Negative atomic charges are localized on oxygen atoms, with metal-coordinated oxygen atoms showing more negative charges compared to non-coordinated oxygen atoms. The comparison demonstrates that Sr induces more negative atomic charges on oxygen atoms relative to Ca, which is consistent with the higher ionic nature of Sr-O bonding.

## 4. Antimicrobial Analysis of SrHAp

**Figure 8:**
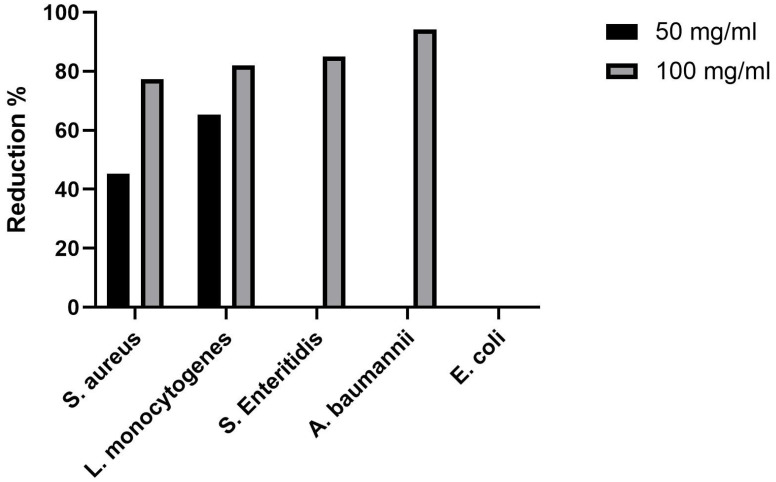
Reduction in viable bacterial cells after the 24 h treatment with SrHAp powder compared with non-treated cells.

The antimicrobial activity of SrHAp material at two different concentrations against five pathogenic microorganisms is shown in Table 5. The number of viable cells of all microorganisms treated with different concentrations of SrHAp decreased compared to the control after 24 h of incubation. It can be seen that the decrease in viable cells was dose-dependent. Compared to an initial number of viable cells (Control 0 h), SrHAp showed no effects. However, compared to the positive control, in which the bacteria were grown without antimicrobial material, an inhibitory effect was observed. Higher concentrations of SrHAp expressed statistically significant inhibitory effect against all tested bacteria except *E. coli.* The number of viable cells of *S. aureus*, *L. monocytogenes*, *S.* Enteritidis, and *A*. *baumanii* was reduced by 77.38%, 82%, 85%, and 94.14%, respectively, compared to the positive control after 24 h of incubation (Figure 8). At lower concentration tested, SrHAp material was effective against both Gram-positive bacterial species, *S. aureus* and *L. monocytogenes*, with a reduction of 45.24% and 65.33% of cells, respectively. Sonication in an ultrasonic bath did not increase the number of viable adherent bacteria, indicating no detectable bacterial adhesion on the powder surface.

To the best of our knowledge, this is the first report that SrHAp material demonstrated activity against *L. monocytogenes*. While primarily recognized as foodborne pathogen, *L. monocytogenes* can survive in harsh environmental conditions and also cause infections in hospitalized individuals, especially in immunocompromised persons, the elderly, pregnant women, and neonatal. In some cases, prosthetic materials have led to bone and joint infections, including listeriosis. This issue can be significantly reduced when biocompatible materials are applied in a thin layer [67]. Previously, various Sr-incorporated HAp materials were the subject of antimicrobial evaluation. In the study by Lin et al. [68], SrHAp containing 53.8% Sr reduced the number of *E. coli* by 91.9% and *S. aureus* by 91.6% at a concentration of 100 mg/mL. In another study, SrHAp coating of TiO_2_ nanotube implants partially reduced *S. aureus* (37.2%) [69]. However, Geng et al. [70] investigated the antibacterial activity using the disk diffusion method and found no activity of the coating against *E. coli* and *S. aureus*. These bacteria were also resistant to hydrothermally synthesized Sr-HAp microspheres [71]. Given its proven ability to inhibit the growth of various bacterial species, the developed SrHAp material may be a promising candidate for controlling microbial contamination across diverse applications.

## 5. Discussion

Various characteristics of nanomaterials such as size, morphology, zeta potential, and charge play an important role in their behavior. Primarily, nanoparticles attach to bacteria’s cell wall enabling further penetration into the cell causing obstruction of metabolic functions [72,73]. Once inside the cells, SrHAp nanocrystals may act through different mechanisms that can be interconnected. It may affect the production of intracellular ATP that may disrupt the process of DNA replication. Also, it may cause excessive generation of reactive oxygen species (ROS) such as hydrogen peroxide (H_2_O_2_) and superoxide-radical O_2_^•−^. When intracellular antioxidant defense system is not able to eliminate all ROS, their concentration increases and consequently causes lethal damages [74,75].

The effective antimicrobial properties of the SrHAp material can be attributed to the interactions between the metal ions in the HAp structure and the functional groups of the proteins comprising bacterial cell membranes [76]. The interactions between materials and biomolecules are dynamic and highly complex, with antimicrobial efficacy strongly correlated with negative structural charges and nanocrystalline morphology, which initiates protein degradation and subsequent degradation of bacterial cells. In this study, the synergistic effect of surface charges and nanoscale crystals enables physical penetration into bacterial cells. Nanomaterials with smaller crystal diameters exhibit increased bacterial toxicity [77]. The synthesized sample exhibits a homogeneous nanorod-like morphology with a size of 5 nm and a high specific surface area, which contributes to antibacterial activity by disrupting the bacterial cell membrane.

In this research, SrHAp material expressed inhibitory activity against both Gram-positive and Gram-negative bacterial species. Lower SrHAp concentrations selectively inhibited the growth of Gram-positive bacteria, which is due to the outer lipopolysaccharide (LPS) layer in Gram-negative bacterial cell membranes. However, higher SrHAp concentrations successfully overcame this defense mechanism and reduced viable Gram-negative bacterial cells by up to 94.14% compared to the positive controls. This effect results from the direct contact between microorganisms and SrHAp nanocrystals. Antimicrobial tests at a pH value of 7 ensured positive surface charge for the SrHAp nanomaterial investigated. Under physiological conditions, proteins acquire a negative charge, with increased incorporation of the metal ions into the doped HAp structure leading to enhanced protein adsorption on SrHAp nanocrystal surfaces. In addition, OH- groups positioned along the c-axis in the hexagonal hydroxyapatite structure (hydroxyl ion channels) provide structural advantages, as chemisorption occurs predominantly along these accessible channels that facilitate the uptake of material into the structure [78].

## 6. Conclusions

This study successfully demonstrates the synthesis and comprehensive characterization of nanocrystalline strontium-doped hydroxyapatite (SrHAp) with antimicrobial properties. X-ray diffraction analysis confirmed the formation of single-phase hexagonal SrHAp with space group symmetry P63/m and unit cell parameters *a* = 9.41 Å and *c* = 6.92 Å. The successful incorporation of Sr^2+^ ions into the Ca^2+^ sites was verified by shifting the lattice parameter and ICP-OES analysis, achieving the targeted partial substitution within the 15% limits with a final composition of Sr_0.7_Ca_4.3_(PO_4_)_3_OH. FTIR spectroscopy revealed characteristic hydroxyapatite bands adjacent to carbonate impurities, confirming structural integrity despite the minor A-type carbonate substitution. Morphological analysis demonstrated uniform nanorod-like structures with a size of 25 × 10 nm and a crystallite size of about 4.9 nm, forming elongated agglomerates of 5–7 μm in length. DFT calculations elucidated the structural differences between Ca-HAp and Sr-HAp, revealing larger Sr-O bond lengths (2.621 Å vs. 2.432 Å for Ca-O) and enhanced ionic character due to the larger Sr^2+^ ionic radius.

The material exhibited a high pH_PZC_ value, with an average of 10, which ensures a positive surface charge at physiological pH conditions. The antimicrobial tests showed a promising inhibitory effect against both Gram-positive and Gram-negative bacteria, with a reduction in viable cells of up to 94.14% at higher concentrations. The enhanced antimicrobial activity results from the synergistic effects of positive surface charge, nanoscale morphology, and direct interaction with the bacterial cell membrane. While these findings highlight SrHAp as a promising candidate for biomedical applications requiring antimicrobial properties, further studies can be conducted to validate them under expanded experimental conditions and larger sample sizes, thereby confirming the material’s applicability and functionality. This will provide insights into its potential effectiveness for controlling microbial contamination in various fields including biomedical, environmental, technological, and material science applications.

## Figures and Tables

**Table 1:** Refined profile and structural and microstructural parameters of SrHAp.

Space Group	Unit Cell Parameters (Å, Å^3^)					Figure of Merit (%)
P6_3_/m	*a*	*b*	*c*	*V*	crystallite sizes	*Rwp* = 6.24
	9.41 (5) Å	9.41 (5) Å	6.92 (8) Å	531 (7) Å^3^	48.8 (8) Å	*S* = 1.5152

**Table 2:** Nominal and experimentally obtained concentrations of elements.

Elements	Nominal Concentrations (%)	ICP OES (%)
Ca	29.11	25.8
Sr	15.94	11.53
P	16.92	17.90

**Table 3:** Molar ratio of elements, nominal and obtained by ICP OES.

Elements	Nominal Ratio	ICP OES Ratio
Ca/P	1.33	1.12
Ca/Sr	4.0	4.8
(Ca + Sr)/P in HAP	1.67	1.35

**Table 4:** Mulliken atomic charges for investigated model systems.

Compound	M (Ca, Sr)	P	O_coord_	O
1. [Ca(PO_4_)_2_]^4−^	1.550850	0.456892	−0.818077, −0.823031, −0.820176	−0.770593
		0.460227	−0.819471, −0.824140, −0.822686	−0.769794
2. [Sr(PO_4_)_2_]^4−^	1.710723	0.405369	−0.823579, −0.821199, −0.824062	−0.791906
		0.405468	−0.821761, −0.823546, −0.823669	−0.791838

**Table 5:** Antimicrobial effect of SrHAp material at two different concentrations expressed as log_10_ CFU/mL.

Bacteria	Inoculum 0 h	Control 24 h	SrHAp 50 mg/mL 24 h	SrHAp 100 mg/mL 24 h
*Staphylococcus aureus*	5.42 ± 0.08 ^a,1^	9.32 ± 0.02 ^b^	8.97 ± 0.03 ^c^	8.68 ± 0.03 ^d^
*Listeria monocytogenes*	5.74 ± 0.06 ^a^	9.87 ± 0.12 ^b^	9.41 ± 0.9 ^c^	9.13 ± 0.16 ^d^
*Salmonella* Enteritidis	5.23 ± 0.60 ^a^	9.30 ± 0.0 ^b^	9.43 ± 0.07 ^b^	8.47 ± 0.0 ^c^
*Acinetobacter baumannii*	5.46 ± 0.04 ^a^	9.45 ± 0.26 ^b^	9.58 ± 0.20 ^b^	8.21 ± 0.13 ^c^
*Escherichia coli*	5.61 ± 0.54 ^a^	9.35 ± 0.21 ^b^	9.54 ± 0.09 ^b^	9.39 ± 0.11 ^b^

^1^ Statistically significant differences (*p* < 0.05) among samples are indicated by different superscript letters in the same column.

## Data Availability

The original contributions presented in this study are included in the article. Further inquiries can be directed to the corresponding author.
